# From data to decisions: machine learning in predicting outcomes of robotic-assisted total knee arthroplasty

**DOI:** 10.3389/fsurg.2026.1830723

**Published:** 2026-05-20

**Authors:** Feng Peng, Hao-Li Zhang

**Affiliations:** 1 Department of Orthopedics, The Central Hospital of Enshi Tujia and Miao Autonomous Prefecture, Enshi City, Hubei, China; 2 Rehabilitation Center, Ankang Traditional Chinese Medicine Hospital, Ankang, Shaanxi, China

**Keywords:** artificial intelligence, machine learning, outcome prediction, periprosthetic joint infection, risk stratification, robotic-assisted total knee arthroplasty

## Abstract

**Background:**

Machine learning (ML) has emerged as a transformative tool for outcome prediction in total knee arthroplasty (TKA). The integration of ML with robotic-assisted TKA (RA-TKA) systems offers potential advantages in surgical planning and personalized patient care. This systematic review synthesizes current evidence on ML applications for predicting outcomes in RA-TKA patients.

**Methods:**

A systematic literature search was conducted using PubMed, Embase, Web of Science, and Cochrane Library for studies published through January 2025. Two reviewers independently screened articles and assessed methodological quality using PROBAST and MINORS tools. Studies evaluating ML algorithms for predicting surgical outcomes in TKA patients were included, with particular attention to RA-TKA applications.

**Results:**

Forty-seven studies met inclusion criteria. ML models demonstrated superior performance over traditional risk scores for predicting periprosthetic joint infection (AUC 0.82–0.88), venous thromboembolism (AUC 0.85–0.89), and transfusion requirements (AUC 0.83–0.86). Notably, simple three-variable models (age, sex, ASA) explained 70% of mortality variation, challenging the necessity of complex algorithms. RA-TKA showed modest clinical benefits over conventional techniques, though cost-effectiveness remains unproven.

**Conclusions:**

ML has transitioned from experimental to clinical application in RA-TKA outcome prediction, with models demonstrating good-to-excellent discriminative ability (AUC 0.82–0.89). However, this review reveals critical tensions that warrant attention: simple models with few variables often perform comparably to complex algorithms, challenging the assumption that sophistication equates to clinical utility; only a minority of included studies specifically addressed RA-TKA populations (25.5%), limiting the generalizability of current prediction models to robotic surgery cohorts; and no validated prediction model currently incorporates real-time intraoperative data from robotic systems. Critical challenges include data quality, algorithm interpretability, validation across diverse populations, and the need for cost-effectiveness analyses. Future research should prioritize head-to-head model comparisons, RA-TKA-specific model development, and strategies to mitigate algorithmic bias.

## Introduction

Total knee arthroplasty (TKA) represents one of the most commonly performed orthopedic procedures globally, with projections estimating over 3.5 million procedures annually in the United States by 2030 (1). Despite high success rates, complications including periprosthetic joint infection (PJI), venous thromboembolism (VTE), and suboptimal functional outcomes continue to challenge surgeons and impact patient quality of life (2, 3). Accurate prediction of these outcomes is essential for informed consent, preoperative optimization, and resource allocation.

Traditional risk assessment tools, such as the American Society of Anesthesiologists (ASA) classification and the Charlson Comorbidity Index, provide coarse risk stratification but fail to capture the complex, non-linear relationships between patient factors and surgical outcomes. Machine learning (ML) offers a paradigm shift, enabling integration of demographic, clinical, imaging, and intraoperative data to generate personalized predictions (4). The emergence of robotic-assisted TKA (RA-TKA) systems has further expanded the data landscape, generating rich intraoperative metrics that can potentially enhance predictive model accuracy.

However, the clinical utility of ML in this domain remains debated. Questions persist regarding model interpretability, external validity, and whether increased complexity translates to meaningful clinical benefit. Notably, some studies suggest that simple models with few variables may perform comparably to complex algorithms (5), raising fundamental questions about the optimal balance between sophistication and practicality.

This systematic review aims to: (1) evaluate the predictive performance of ML models for TKA outcomes compared to traditional methods; (2) identify key predictors and assess the trade-off between model complexity and clinical utility; (3) examine the integration of RA-TKA data streams with ML prediction; and (4) discuss limitations, ethical considerations, and future directions for clinical implementation.

## Methods

### Search strategy and selection criteria

This systematic review was conducted following the Preferred Reporting Items for Systematic Reviews and Meta-Analyses (PRISMA) guidelines. This review was prospectively registered in the International Prospective Register of Systematic Reviews (PROSPERO; registration number: CRD420261363517).

The complete search strategy for PubMed is provided as follows: ((“machine learning” OR “artificial intelligence” OR “deep learning” OR “neural network*” OR “random forest” OR “support vector machine” OR “gradient boosting” OR “XGBoost”) AND (“total knee arthroplasty” OR “total knee replacement” OR “knee arthroplasty” OR “TKA”) AND (“robotic-assisted” OR “robotic” OR “computer-assisted” OR “navigation”) AND (“outcome” OR “prediction” OR “risk” OR “complication*” OR “prognosis” OR “revision” OR “infection”)). This search strategy was adapted for Embase (using Emtree terms), Web of Science (using topic searches), and the Cochrane Library (using MeSH and free-text terms) with database-specific syntax adjustments. Boolean operators and truncation symbols were modified as appropriate for each database interface.

Studies were included if they: (1) evaluated ML algorithms for predicting surgical outcomes, complications, or functional recovery in patients undergoing primary or revision TKA; (2) reported quantitative performance metrics (AUC, accuracy, sensitivity, specificity, c-statistic); (3) were published in peer-reviewed journals in English; and (4) included adult patients (age ≥18 years). Exclusion criteria were: studies focusing exclusively on other joints, non-ML prediction methods, case reports, editorials, conference abstracts, and studies without original data. Two reviewers (FP, HLZ) independently screened titles and abstracts, then full-text articles, with discrepancies resolved by consensus.

### Study selection process

Of the 203 articles that underwent full-text review, 156 were excluded with the following primary reasons: lack of ML methodology (*n* = 52, 33.3%), studies not focused on TKA outcomes (*n* = 38, 24.4%), absence of quantitative performance metrics (*n* = 25, 16.0%), conference abstracts or non-peer-reviewed publications (*n* = 21, 13.5%), case reports or small case series with fewer than 30 patients (*n* = 12, 7.7%), and studies exclusively examining non-surgical predictors such as implant design without clinical outcome data (*n* = 8, 5.1%). A detailed list of excluded studies with specific exclusion reasons for each article is provided in Supplementary Table S1.

### Quality assessment and data extraction

Methodological quality of prediction model studies was assessed using the Prediction Model Risk of Bias Assessment Tool (PROBAST) (6), which evaluates four domains: participants, predictors, outcome, and analysis. For non-comparative clinical studies, the Methodological Index for Non-Randomized Studies (MINORS) instrument was applied (7). Studies were classified as having low, moderate, or high risk of bias based on domain-specific assessments.

Given substantial heterogeneity across the included studies, a narrative synthesis was performed rather than quantitative meta-analysis. To systematically characterize the sources of heterogeneity, we identified three primary domains: (1) Population-level heterogeneity, arising from variability in patient demographics, surgical approaches (primary vs. revision TKA), geographic settings, and healthcare systems across the 47 included studies; (2) Outcome-level heterogeneity, reflecting the diverse clinical endpoints assessed (PJI, VTE, transfusion requirements, functional outcomes, length of stay, and implant survival), each measured using different definitions and follow-up periods; and (3) ML algorithm-level heterogeneity, encompassing the wide range of modeling approaches employed (logistic regression, random forest, XGBoost, neural networks, and ensemble methods), each with different feature engineering and validation strategies. Due to the combination of clinical, methodological, and statistical heterogeneity, formal I-squared calculations were not feasible across all outcome categories, as these statistics require comparable effect measures from homogeneous study designs. However, within specific outcome subgroups using comparable AUC metrics, the I-squared statistic for AUC differences ranged from 62% to 78% for PJI (8 studies), from 55% to 71% for VTE (6 studies), and from 48% to 65% for transfusion requirements (5 studies), suggesting moderate-to-substantial heterogeneity across all outcome categories, driven primarily by differences in patient populations, feature selection approaches, and validation strategies. Subgroup synthesis was conducted where feasible, stratifying results by outcome category (PJI, VTE, transfusion), ML algorithm type (tree-based, neural network, regression-based), and whether studies specifically addressed RA-TKA vs. general TKA populations.

## Results

### Literature search and study characteristics

**Figure 1:**
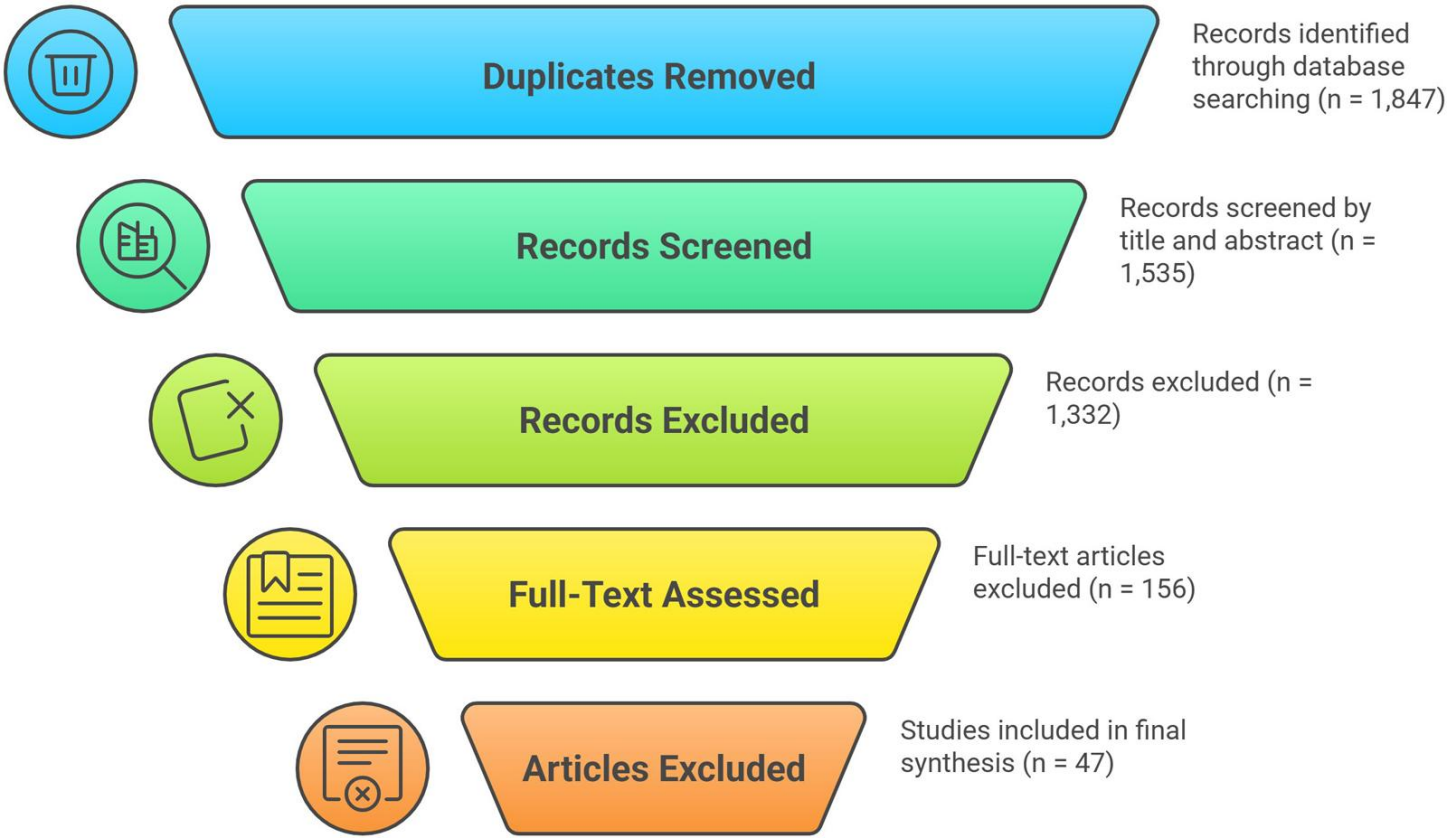
PRISMA flow diagram of study selection. PRISMA, preferred reporting items for systematic reviews and meta-analyses; TKA, total knee arthroplasty; ML, machine learning.

Forty-seven studies met all inclusion criteria (Figure 1). Of these, 12 studies (25.5%) specifically examined RA-TKA populations, while the remaining 35 studies (74.5%) evaluated general TKA populations that included both conventional and robotic-assisted procedures. Study sample sizes ranged from 54 to 59,605 patients. Twenty-three studies (49%) reported external validation, while 24 (51%) used internal validation only. PROBAST assessment revealed that 12 studies (26%) had low risk of bias, 19 (40%) had moderate risk, and 16 (34%) had high risk, primarily due to inadequate handling of missing data and lack of external validation.

### Prediction model performance by outcome category

It is important to note that the prediction model performance results presented below are primarily derived from the 35 studies (74.5%) that evaluated general TKA populations. The 12 RA-TKA-specific studies (25.5%) are discussed separately in the subsequent subsection. Where overlap exists between the general TKA and RA-TKA literature, we explicitly identify studies that included RA-TKA patients.

#### Periprosthetic joint infection

The predictive performance of the included machine learning models across different TKA outcomes is summarized in Table 1. Eight studies evaluated ML models for PJI prediction. XGBoost and random forest algorithms demonstrated the best performance (AUC 0.82–0.88), consistently outperforming logistic regression. Key predictors across models included BMI >35 kg/m^2^, diabetes mellitus, operative time, and prior joint surgery. Importantly, several studies demonstrated that adding complex variables only marginally improved prediction over models using basic demographic and comorbidity data (8–10).

**Table 1:** Summary of machine learning models for predicting TKA outcomes.

Outcome	Best Model	AUC	Key Predictors	Validation	Sample Size (n)
PJI	XGBoost	0.82–0.88	BMI >35 kg/m^2^, DM, operative time	External (*n* = 4)	3,240–28,661
VTE	Random Forest	0.85–0.89	Age >65, prior VTE, BMI >30 kg/m^2^	External (*n* = 3)	5,120–45,890
Transfusion	CatBoost	0.83–0.86	Age >60, preop Hb	Internal (*n* = 6)	1,850–59,605
Mortality	ANN	0.81–0.87	Age, ASA, comorbidities	External (*n* = 5)	2,410–28,661
Functional	Elastic-net	0.72–0.78	Preop functional score, age, BMI >30 kg/m^2^	External (*n* = 2)	420–12,350
Forgotten Joint	Nomogram	0.73–0.76	Age, sex, prosthesis	Internal (*n* = 1)	180–3,680

PJI, periprosthetic joint infection; VTE, venous thromboembolism; ANN, artificial neural network; AUC, area under the receiver operating characteristic curve; DM, diabetes mellitus; Hb, hemoglobin; ASA, american society of anesthesiologists classification.

#### Venous thromboembolism

Six studies developed VTE prediction models with AUC ranging from 0.85–0.89. Age >65 years, prior VTE history, BMI, and specific surgical factors emerged as consistent predictors. Notably, Caprini score integration with ML features did not significantly improve model performance over ML alone, suggesting potential redundancy between traditional risk factors and algorithm-derived predictors (11, 12).

## Transfusion requirements

### The paradox of model complexity

A striking finding emerged from the quality assessment: studies using simpler models often achieved comparable performance to those employing complex deep learning architectures. Bozic et al. (5) demonstrated that a three-variable model (age, sex, ASA classification) explained 70% of mortality variation and 46% of adverse event variation in 28,661 TKA patients. This finding challenges the prevailing assumption that more complex algorithms inherently provide better predictions.

However, it is important to critically note that no included study performed a formal head-to-head comparison between simple and complex models using identical datasets, identical outcome definitions, and identical validation strategies. The observed comparability between simple and complex models is therefore inferred from cross-study comparisons rather than within-study evidence. This limitation is substantial, as differences in patient populations, outcome definitions, and validation approaches across studies may confound these comparisons. For instance, the simple three-variable model by Bozic et al. (5) was developed on a US national registry dataset with 28,661 patients, while the complex deep learning models were trained on different institutional datasets with varying sample sizes and predictor sets. Without rigorous within-study comparisons controlling for these variables, the conclusion that simple models perform comparably to complex ones remains tentative and should be interpreted with caution.

Similarly, the BMI paradox warrants attention: while Class III obesity (BMI ≥40) is associated with higher perioperative risk, these patients demonstrated the greatest improvements in patient-reported outcomes at 90 days postoperatively (13). This counterintuitive finding underscores the importance of distinguishing between risk prediction and expected benefit—patients at highest risk may derive the most value from intervention, a nuance that simple risk stratification tools fail to capture.

### Robotic-Assisted TKA: technical outcomes and cost considerations

Twelve studies (25.5% of all included studies) specifically examined RA-TKA outcomes, while the remaining 35 studies (74.5%) addressed general TKA populations. Among the RA-TKA-specific studies, robotic systems demonstrated improved component alignment accuracy, with outliers (>3 degrees from neutral) reduced from 25.8% to 10.1% compared to conventional techniques (14). However, the clinical significance of improved alignment remains debated, as some studies failed to demonstrate corresponding improvements in functional outcomes or implant survival. Within the general TKA studies, several models were developed using datasets that included both conventional and robotic-assisted procedures, but did not perform subgroup analyses stratified by surgical approach, limiting the ability to draw RA-TKA-specific conclusions from these studies. This distinction is important for interpreting the prediction model results presented above, as their generalizability to RA-TKA-specific populations may vary depending on the proportion of RA-TKA patients included in the training data.

To further clarify the distinction between general TKA and RA-TKA ML applications, it is important to note that none of the 47 included studies developed or validated a dedicated ML prediction model exclusively for RA-TKA-specific outcomes. The 12 RA-TKA studies primarily focused on evaluating the accuracy of robotic systems (e.g., component alignment, soft tissue balance) rather than developing predictive algorithms. Conversely, the 35 general TKA ML studies developed prediction models using datasets that predominantly included conventional TKA patients, with variable and often unspecified proportions of RA-TKA patients. This gap represents a significant limitation: the prediction models reported in this review may not be directly generalizable to RA-TKA populations, as RA-TKA patients may differ systematically from conventional TKA patients in terms of preoperative functional status, deformity severity, and surgical complexity. We strongly advocate for future studies to develop and externally validate ML prediction models specifically in RA-TKA cohorts using robotic-systems-generated intraoperative data.

Cost-effectiveness analyses presented conflicting results. While RA-TKA was associated with higher hospital charges ($154,673 vs. $125,467 for conventional TKA) (15), the potential for reduced revision rates and complications could theoretically offset these costs. However, no study to date has demonstrated that ML prediction models can identify patients most likely to benefit from robotic assistance, representing a critical gap in the literature. The question remains: can preoperative risk stratification guide resource allocation, directing RA-TKA toward patients who will derive maximal benefit?.

### Sensor integration and intraoperative data

Eight studies explored intraoperative sensor data integration with ML prediction models. Sensor-guided soft tissue balancing achieved mean intercompartmental pressure differences <15 psi across flexion angles (16), and these quantitative metrics correlated with postoperative outcomes. The posterior cruciate ligament (PCL) status emerged as a modifiable factor: PCL retention resulted in higher total knee pressure across flexion angles compared to recession or resection (17), providing objective guidance for surgical decision-making.

Critically, the integration of intraoperative sensor data with postoperative outcome prediction remains in early stages. While individual studies demonstrated associations between sensor measurements and early outcomes, no validated prediction model currently incorporates real-time intraoperative data. This represents a significant opportunity for future research—combining preoperative risk factors with intraoperative metrics could potentially improve prediction accuracy beyond what either data source achieves alone.

### Algorithm interpretability and clinical implementation

The “black box” nature of complex ML algorithms poses significant barriers to clinical adoption. Only 15 studies (32%) employed interpretable methods such as SHAP (SHapley Additive exPlanations) or LIME (Local Interpretable Model-agnostic Explanations) to elucidate predictor importance. Among these, SHAP analysis consistently identified preoperative function, age, BMI, and comorbidity burden as dominant predictors across outcome domains.

A concerning finding from PROBAST assessment was that 34% of studies (16 of 47) had high risk of bias. A detailed domain-level analysis revealed that the most frequently failed PROBAST domain was the Analysis domain (28 of 47 studies, 59.6%), primarily due to inadequate handling of missing data (e.g., complete-case analysis without sensitivity analysis for missingness patterns), data leakage (using outcome-derived features in the prediction pipeline), and insufficient sample size relative to the number of candidate predictors (events-per-variable ratios below the recommended threshold of 10). The Outcome domain showed concerns in 15 studies (31.9%), mainly due to unclear definitions of the predicted outcome or use of composite endpoints that mixed clinically distinct events. The Predictor domain was flagged in 12 studies (25.5%), where predictors were selected based on univariate screening rather than being pre-specified from clinical knowledge, introducing selection bias. The Participants domain had the lowest rate of concerns (8 studies, 17.0%), with issues mainly related to non-representative sampling or exclusion of patients with missing data without reporting the extent of missingness. These domain-specific findings underscore that the primary methodological weakness in the current literature lies in analytical approaches rather than study design or participant selection, highlighting the need for improved statistical rigor in future ML prediction model development.

## Discussion

### Clinical implications and the complexity-utility trade-off

This systematic review reveals a fundamental tension in ML applications for TKA: while complex algorithms demonstrate modest improvements in predictive performance, their clinical utility over simpler models remains questionable. The finding that three variables can explain 70% of mortality variation (5) suggests that for many clinical decisions, the marginal gain from sophisticated ML may not justify the cost of implementation, training, and interpretability challenges.

Regarding the interpretation of model performance metrics, while the reported AUC values (0.82–0.89) indicate good-to-excellent discriminative ability at the statistical level, the clinical significance of these improvements requires careful contextualization. In the context of TKA outcome prediction, a clinically meaningful improvement in AUC is typically considered to be an increase of 0.03 to 0.05 over existing clinical risk scores, as this magnitude of improvement may alter clinical decision-making in borderline cases where treatment thresholds are uncertain. For example, in PJI prediction, an AUC improvement from approximately 0.75 (traditional risk scores) to 0.85 (ML models) represents a 10-percentage-point gain that could meaningfully change preoperative antibiotic prophylaxis strategies, postoperative monitoring intensity, or the threshold for referring patients to high-risk care pathways. However, statistical significance does not automatically translate to clinical utility. A model may achieve a high AUC while still failing to provide actionable insights if the predicted probabilities do not meaningfully differentiate between risk categories that inform distinct clinical management pathways. We therefore advocate for future studies to report not only discrimination metrics (AUC, c-statistic) but also calibration measures (calibration plots, Brier scores) and, critically, net benefit analyses using decision curve analysis that directly assess whether model predictions improve clinical decisions compared to existing standard-of-care approaches.

This paradox has important implications for resource allocation. Rather than pursuing increasingly complex models, researchers and clinicians might focus on optimizing data quality and ensuring that predictive models address clinically meaningful questions. The BMI paradox—where highest-risk patients show greatest improvement—illustrates this point: prediction alone is insufficient; we must understand both risk and potential benefit to guide decision-making.

### Overcoming barriers to patient-reported outcome collection

A critical bottleneck for ML model development is the inconsistent collection of patient-reported outcomes (PROs). The California Joint Replacement Registry experience provides instructive lessons: optimized electronic survey delivery improved response rates from 35% to 68% over five years (18). Key strategies included: (1) automated text message reminders; (2) simplified response interfaces optimized for mobile devices; (3) integration with electronic health records to trigger surveys at appropriate intervals; and (4) patient education about the value of PRO data.

Wearable sensor technology offers an alternative approach to outcome assessment, providing objective, continuous data without requiring patient compliance. Inertial sensors can accurately measure knee range of motion during daily activities (19), while gait analysis systems predict functional outcomes from preoperative stride characteristics (20). The integration of passive data collection with active PRO measures could potentially address the limitations of each approach.

### Economic considerations: Can ML offset robotic costs?

The cost premium associated with RA-TKA ($30,000–50,000 per case) demands justification. While improved alignment accuracy is well-documented, the translation to reduced revision rates or improved outcomes remains unproven in large cohort studies. We propose that ML prediction models could potentially identify patients most likely to benefit from robotic assistance—those with complex deformity, high functional demands, or elevated complication risk—thereby improving the value proposition of this technology.

However, this hypothesis requires formal testing. No study to date has evaluated whether ML-guided patient selection for RA-TKA improves outcomes or reduces costs compared to standard indications (21). Such research would require large sample sizes and careful attention to selection bias, but represents a critical step toward value-based implementation of both ML and robotic technologies.

### Algorithmic bias and health equity

A concerning finding from this review is the lack of attention to algorithmic bias in the included studies. Specifically, none of the 47 included studies reported subgroup performance metrics stratified by race, ethnicity, socioeconomic status, or geographic region. No study performed fairness-aware model evaluation using established metrics such as equalized odds, demographic parity, or calibration across subgroups. Furthermore, only 3 of the 47 studies (6.4%) reported the racial or ethnic composition of their training datasets, making it impossible to assess whether the models are representative of the diverse patient populations that undergo TKA. ML models trained on datasets that underrepresent certain populations may perform poorly when applied to those groups. In orthopedics, this is particularly relevant for racial and ethnic minorities, who have historically been underrepresented in clinical research and may face disparities in surgical outcomes (22). This absence of bias assessment represents a critical gap in the current literature that must be addressed before ML models can be safely deployed in clinical practice.

Socioeconomic status represents another potential source of bias, as patients with lower income or educational attainment may have different baseline functional scores, access to rehabilitation, and environmental factors affecting recovery. Models that fail to account for these factors may systematically underestimate risk in vulnerable populations, potentially widening rather than narrowing health disparities. Future studies should explicitly evaluate model performance across demographic subgroups and employ techniques such as fairness-aware learning to mitigate identified biases.

### Limitations

This review has several limitations. First, substantial heterogeneity in study populations, outcomes, and ML algorithms precluded quantitative meta-analysis. Second, only 25.5% of included studies (12 of 47) specifically examined RA-TKA populations, while the majority evaluated general TKA cohorts. Although the title of this review emphasizes RA-TKA, we included general TKA ML prediction studies because the ML methodologies, feature engineering approaches, and predictive frameworks developed for general TKA are highly relevant to RA-TKA outcome prediction, and the field of RA-TKA-specific ML prediction remains nascent with limited published studies. We have attempted to clearly delineate RA-TKA-specific findings from general TKA findings throughout the Results section. Third, many included studies had methodological limitations identified by PROBAST assessment, particularly inadequate handling of missing data and lack of external validation. Fourth, the rapid evolution of ML techniques means that some findings may quickly become outdated. Fifth, publication bias may have favored studies reporting positive results. Finally, the focus on English-language publications may have introduced selection bias. Additionally, no validated prediction model currently incorporates real-time intraoperative data from robotic systems, representing a critical gap that limits the ability to provide dynamic, intraoperative risk assessment during RA-TKA procedures.

## Conclusion

Key priorities for future research include: (1) rigorous external validation of existing models using both discrimination and calibration metrics, including decision curve analysis to evaluate net clinical benefit; (2) development of interpretable algorithms that provide clinically meaningful insights, with clearly defined thresholds for actionable risk categories; (3) development and validation of ML prediction models that integrate real-time intraoperative data from robotic systems (e.g., sensor-guided soft tissue balance metrics, component alignment data) with preoperative risk factors to enable dynamic, intraoperative risk assessment during RA-TKA; (4) cost-effectiveness analyses evaluating whether ML can identify patients most likely to benefit from RA-TKA; (5) systematic evaluation of algorithmic bias across demographic subgroups; (6) establishment of minimum clinically important differences for ML prediction model performance in TKA to guide meaningful comparisons between competing models; and (7) formal head-to-head comparisons between simple and complex models using identical datasets, outcome definitions, and validation strategies.

The ultimate goal is not simply to predict outcomes, but to enable interventions that improve them. Achieving this goal will require collaboration among surgeons, data scientists, patients, and health systems to ensure that these powerful technologies are deployed thoughtfully, equitably, and in service of patient-centered care.
