# Supplementary material for: From data to decisions: machine learning in predicting outcomes of robotic-assisted total knee arthroplasty
Source: Front Surg. 2026 May 20;13:1830723. doi: 10.3389/fsurg.2026.1830723 (PMC13230051; doi:10.3389/fsurg.2026.1830723)
Supplement: Supplementary file 1 [file Table1.docx]

**Table S1 Exclusion Criteria and Codes**

| **Code** | **Category** | **Description** |
| --- | --- | --- |
| **E1** | No ML method | Studies entirely lacking ML/AI methods |
| **E2** | Non-TKA | Studies on THA, UKA, or other procedures |
| **E3** | No prediction/outcome model | Only image segmentation etc., without clinical outcome prediction |
| **E4** | Insufficient performance metrics | No quantitative metrics such as AUC or accuracy reported |
| **E5** | Conference abstract/insufficient data | Published as abstract only, quality cannot be assessed |
| **E6** | Overlapping patient cohorts | Shared datasets with included studies |
| **E7** | Non-English literature | Full text unavailable in English |
| **E8** | Full text inaccessible | Full text could not be obtained |
